# Clinical manifestations and outcomes of idiopathic ventricular fibrillation and early repolarization syndrome in adolescents: A multicenter cohort study

**DOI:** 10.1016/j.hroo.2026.02.016

**Published:** 2026-02-24

**Authors:** Seung Min Baek, Jae Suk Baek, Joowon Lee, Ja Kyoung Yoon, Hye Won Kwon, Young Hye Ryu, So Yon Jun, Mi Jin Kim, Ah Young Kim, Chang Sin Kim, Ji Eun Ban, June Huh, Eun Jung Bae, Jae-Sun Uhm, Mi Kyoung Song

**Affiliations:** 1 Department of Pediatrics, Seoul National University Children's Hospital, Seoul, Republic of Korea; 2 Department of Pediatrics, Asan Medical Center, University of Ulsan College of Medicine, Seoul, Republic of Korea; 3 Department of Pediatrics, Seoul National University Bundang Hospital, Bundang, Republic of Korea; 4 Department of Pediatrics, Samsung Medical Center, Sungkyunkwan University School of Medicine, Seoul, Republic of Korea; 5 Department of Thoracic and Cardiovascular Surgery, Seoul National University Children's Hospital, Seoul, Republic of Korea; 6 Department of Pediatrics, Severance Cardiovascular Hospital, Yonsei University College of Medicine, Seoul, Republic of Korea; 7 Department of Pediatrics, Sejong General Hospital, Bucheon, Gyeonggi-do, Republic of Korea; 8 Division of Cardiology, Department of Internal Medicine, Severance Cardiovascular Hospital, Yonsei Univerty College of Medicine, Seoul, Republic of Korea

**Keywords:** Idiopathic ventricular fibrillation, Early repolarization syndrome, J-wave syndrome, Pediatrics, Arrhythmias

## Abstract

**Background:**

Idiopathic ventricular fibrillation (IVF) and early repolarization syndrome (ERS) are rare but potentially life-threatening arrhythmias. Their clinical distinction is often challenging and prognostic factors remain unclear, particularly in adolescents.

**Objective:**

This study compared clinical outcomes and electrocardiographic (ECG) characteristics of adolescents with IVF and ERS to identify disease-specific prognostic factors.

**Methods:**

Patients diagnosed as having IVF or ERS between 2000 and 2022 were retrospectively reviewed from a nationwide Korean multicenter cohort of pediatric inherited arrhythmia syndromes. ECGs were systematically reanalyzed for early repolarization patterns (ERPs). Life-threatening arrhythmic events (LAEs), including ventricular arrhythmias, appropriate implantable cardioverter-defibrillator shock, and sudden cardiac arrest, were compared between the groups.

**Results:**

ECG reanalysis led to the reclassification of 10 patients from IVF to ERS, resulting in 14 patients with ERS and 25 patients with IVF (mean age 16.0 ± 2.3; 92.3% male). LAEs were more frequent in the ERS group (92.9% vs 52.0%; *P* = .025), with shorter LAE-free survival (median 7.2 vs 51.6 months; *P* = .005). In ERS, inferolateral ERP predicted poorer prognosis than inferior ERP (hazard ratio 9.29 [1.68–101.36]; *P* = .009), whereas coexistence of supraventricular tachycardia was a risk factor for LAEs in IVF (hazard ratio 7.24 [1.89–29.73]; *P* = .005). J-wave duration was longer in inferolateral ERP and in cases with pause-dependent augmentation.

**Conclusion:**

IVF and ERS have distinct prognoses and risk factors in adolescent patients. ECG-based differentiation and identification of prognostic factors may guide individualized strategies.

## Graphical abstract

**Figure:**
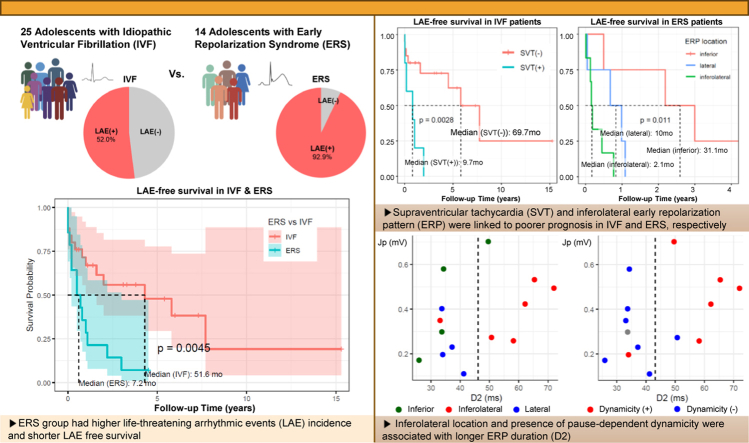

**Key Findings**

▪ Early repolarization syndrome (ERS) showed a higher recurrence of life-threatening arrhythmic events (LAEs) and shorter LAE-free survival than idiopathic ventricular fibrillation (IVF) in adolescents.
▪ Both ERS and IVF in adolescents exhibited higher LAE recurrence rates than those reported in adult patients with the same diagnoses.
▪ In adolescents with ERS, early repolarization pattern (ERP) location is a key prognostic factor, whereas in IVF, supraventricular tachycardia is the strongest predictor of recurrent LAEs.
▪ In ERS, ERP duration, pause-dependent dynamicity, and location are interrelated and collectively influence prognosis.

## Introduction

Idiopathic ventricular fibrillation (IVF) has historically encompassed all cases of sudden cardiac arrest (SCA) with ventricular fibrillation (VF) in structurally normal hearts. However, the identification of genetic arrhythmia syndromes such as long QT syndrome, short QT syndrome, Brugada syndrome, catecholaminergic polymorphic ventricular tachycardia, and short-coupled VF^1^ has redefined IVF and clarified its mechanisms. Similarly, early repolarization pattern (ERP), once regarded as a benign electrocardiographic (ECG) variant, has been reported with a markedly higher prevalence among VF survivors,^2^^,^^3^ leading to the recognition of early repolarization syndrome (ERS) as a distinct genetic arrhythmia syndrome.

Despite these advances, much remains unknown regarding IVF in the pediatric population. In addition, ERPs are often underrecognized,^4^ particularly in pediatric practice, where the widespread recognition of benign juvenile ERPs may hinder the detection of truly pathologic ERPs. Such misconceptions can delay the recognition and treatment of ERS, thereby worsening outcomes.

In this context, comparing the clinical and ECG features of IVF and ERS is essential for improving diagnosis and outcomes. This study analyzed adolescent patients with IVF and ERS from a nationwide Korean multicenter cohort of pediatric inherited arrhythmia syndromes, compared their clinical characteristics, and identified prognostic factors specific to each.

## Methods

### Study population and data collection

We included patients from the nationwide Korean multicenter cohort of pediatric inherited arrhythmia syndromes, who were initially diagnosed as having ERS or IVF before 19 years of age between 2000 and 2022. Given that the initial diagnoses were not always definitive, the research team systematically reviewed all available ECGs throughout the cohort period to refine diagnoses. Patients with identifiable causes of VF (eg, genetic arrhythmia syndromes, genetic cardiomyopathies, and structural heart diseases) or insufficient clinical data were excluded.

ERS was defined as an unexplained SCA with documented VF or polymorphic ventricular tachycardia, accompanied by ERP (defined below) on 12-lead ECGs. IVF was defined as unexplained VF after excluding other identifiable causes, including ERS. Clinical features including the coexistence of supraventricular tachycardia (SVT) were collected. Family history included unexplained SCA or syncope in first-degree relatives. Functional tests, imaging studies, and genetic testing (including type and results) were collected. During follow-up, we collected data on neurologic sequelae, ventricular arrhythmias, and SCA.

### ECG and ERP

**Figure 1:**
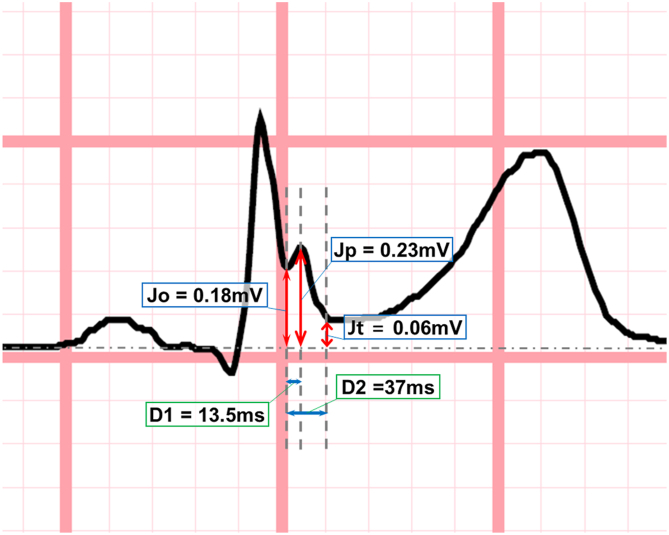
Example of an electrocardiogram from a patient in the cohort (patient identification number 2), illustrating the measurement of J-wave amplitude and duration according to the published consensus. Jo denotes the onset of the J wave, and Jp and Jt represent the amplitudes at the peak and terminal ends of the J wave, respectively. D1 and D2 indicate the durations from Jo to Jp and from Jo to Jt, respectively.

All available ECGs obtained during the follow-up period were reviewed to ensure accurate identification of ERP, given its variable expression over time.^3^ ERP was defined as an end-QRS notch or slur on the downslope of an R wave (amplitude >0.1 mV in at least 2 contiguous leads, excluding V1–V3). ERP amplitude, duration, and ST-segment slope were analyzed from the ECG showing the most prominent ERP, following the consensus guidelines^5^ (Figure 1). 2 pediatric cardiologists (S.M.B. and M.K.S.) independently measured amplitude and duration. For end-QRS notch, amplitude was measured at onset (Jo), peak (Jp), and termination (Jt); duration was Jo–Jp (D1) and Jo–Jt (D2). For end-QRS slur, amplitude was measured at the onset (Jp) and termination (Jt); duration was Jp–Jt (D2). ERP location was classified as inferior (II, III, and aVF), lateral (I, aVL, V4–V6), or inferolateral (both). Pause-dependent dynamicity (Supplemental Figure 1) was recorded when a sudden prolongation of the R-R interval was accompanied by an increase in Jp compared with the preceding beats on serial ECGs or Holter monitor.^6^ Associations among dynamicity, ST-segment slope, ERP location, and ERP amplitude/duration were assessed. Fragmented QRS complex (fQRS) was defined as ≥4 spikes in 1 lead or >1 notch in the nadir of R or S wave in 2 consecutive leads.

### Outcomes

The primary outcome was life-threatening arrhythmic events (LAEs), defined as ventricular tachycardia, VF, appropriate implantable cardioverter-defibrillator (ICD) shocks, and SCA. LAE-free survival was compared between the IVF and ERS groups. Predictors of LAE-free survival were analyzed in each group. For patients with ICDs, appropriate and inappropriate ICD shocks and their dates were recorded.

### Statistical analysis

Data were presented as frequencies, medians with interquartile ranges (IQRs), or means with standard deviations. Group comparisons used χ^2^ test, *t* test, or Wilcoxon rank-sum test, with significance set at *P* < .05. Interobserver agreement for continuous ECG parameters (eg, D1, D2, Jp, and Jt) was assessed using intraclass correlation coefficients (2-way random-effects, absolute agreement, single measurements). Firth’s penalized Cox regression was applied to identify factors associated with LAE-free survival owing to the small group sizes. Variables with *P* < .2 in univariable analysis were included in the multivariable model, with *P* < .05 considered significant. Significant variables and known prognostic factors^7^ were further analyzed using Kaplan–Meier curves and log-rank test. Analyses were performed using SPSS 24.0 (IBM Corp, Armonk, NY) and R 3.4.0 (R Foundation, Vienna, Austria) via R Studio.

### Ethics statement

This study was approved by the institutional review boards of the 6 participating centers and was conducted in accordance with the principles outlined in the Declaration of Helsinki. Owing to the retrospective nature of the study and the absence of identifiable patient data, the institutional review boards waived the requirement for a patient informed consent and the need for review by a critical event committee.

## Results

### Clinical features

**Figure 2:**
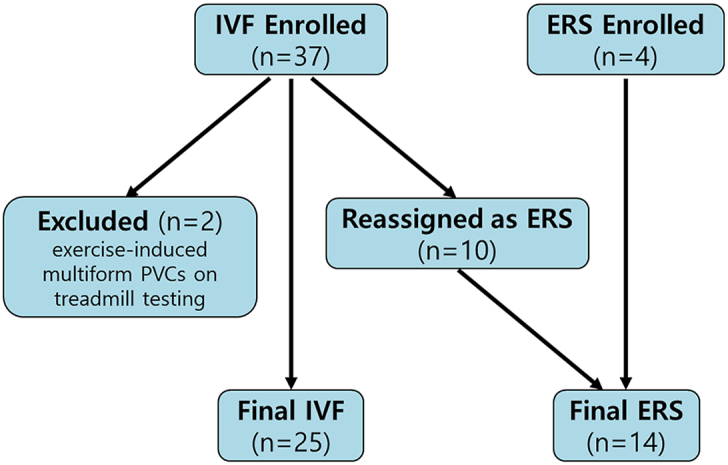
Patient selection and final classification. Of 37 patients initially enrolled as having IVF, 2 were excluded owing to exercise-induced multiform PVCs on treadmill testing. Based on a detailed review of 12-lead electrocardiograms, 10 patients were reassigned to the ERS group. The final cohort included 25 patients with IVF and 14 patients with ERS. ERS = early repolarization syndrome; IVF = idiopathic ventricular fibrillation; PVC = premature ventricular contraction.

The diagnostic reclassification process is presented in Figure 2. Of the 37 patients initially enrolled for IVF, 10 were reassigned to ERS and 2 were excluded owing to exercise-induced multiform premature ventricular contractions on treadmill testing, suggestive of catecholaminergic polymorphic ventricular tachycardia. Finally, 25 patients with IVF and 14 patients with ERS were included.

All 39 patients (92.3% male) presented with VF as the initial manifestation, most (82.1%) without previous symptoms (Table 1). Family history of SCA or unexplained syncope was noted in 10 patients (25.6%), including 2 siblings—1 with IVF and the other with ERS. SVT was identified in 11 patients; all were clinical tachyarrhythmias, except for 1 patient with ERS with atrioventricular reentrant tachycardia induced during an electrophysiological study. No significant clinical differences were observed between the 2 groups.

**Table 1:** Clinical characteristics and diagnostic findings of the study population

Variables			Total	IVF	ERS	*P* value
			(N = 39)	(n = 25)	(n = 14)	
Clinical characteristics	Sex	Male	36 (92.3%)	23 (92.0%)	13 (92.9%)	1.000
	Age at diagnosis (y)		16.0 ± 2.3	16.4 ± 2.4	15.3 ± 2.2	.191
	Triggering circumstance of index VF	Exercise	10 (25.6%)	6 (24.0%)	4 (28.6%)	.852
		Fight	1 (2.6%)	0 (0.0%)	1 (7.1%)	
		Emotional excitation	3 (7.7%)	2 (8.0%)	1 (7.1%)	
		Resting	8 (20.5%)	6 (24.0%)	2 (14.3%)	
		Sleeping	5 (12.8%)	3 (12.0%)	2 (14.3%)	
		Others	2 (5.1%)	1 (4.0%)	1 (7.1%)	
		NA	10 (25.6%)	7 (28.0%)	3 (21.4%)	
	Previous episodes	None	32 (82.1%)	21 (84.0%)	11 (78.6%)	.574
		Syncope	6 (15.4%)	3 (12.0%)	3 (21.4%)	
		Seizure	1 (2.6%)	1 (4.0%)	0 (0.0%)	
	Family history		10 (25.6%)	5 (20.0%)	6 (42.9%)	.250
	SVT during follow-up∗		11 (28.2%)	5 (20.0%)	6 (42.9%)	.156
	SVT type	AF	5 (45.5%)	2 (40.0%)	3 (50.0%)	.329
		Sustained AT	3 (27.3%)	2 (40.0%)	1 (16.7%)	1
		AF and sustained AT	1 (9.1%)	0 (0.0%)	1 (16.7%)	.359
		AVRT	2 (18.2%)	1 (20.0%)	1 (16.7%)	1.000
Diagnostic tests	ECG∗		39 (100%)	25 (100%)	14 (100%)	1
	Heart rate (beats/min)		80.1 ± 23.3	82.3 ± 24.7	79.7 ± 20.6	.740
	QTc (ms)		418.5 ± 29.5	419.4 ± 32.3	417.1 ± 25.3	.814
	Fragmented QRS		25 (64.1%)	18 (75.0%)	7 (46.7%)	.147
	Holter∗		27 (69.2%)	15 (60.0%)	12 (85.7%)	.191
	Average heart rate		75.5 ± 15.6	74.7 ± 15.5	76.8 ± 16.6	.766
	Minimum heart rate		51.2 ± 11.6	47.7 ± 8.0	56.2 ± 14.4	.090
	Maximum heart rate		137.7 ± 31.2	136.3 ± 31.8	139.8 ± 32.2	.805
	Treadmill test∗		22 (56.4%)	11 (44.0%)	11 (78.6%)	.08
	PVC during exercise		1 (4.3%)	0 (0.0%)	1 (9.1%)	.965
	PVC at recovery		1 (4.3%)	1 (8.3%)	0 (0.0%)	.366
	Drug test∗		24 (61.5%)	15 (62.5%)	9 (60.0%)	1.000
	Electrophysiological study∗		12 (30.8%)	7 (28.0%)	5 (35.7%)	.889
	Noninduced		4 (33.3%)	2 (28.6%)	2 (40.0%)	.212
	Induced VT		1 (8.3%)	0 (0.0%)	1 (20.0%)	
	Induced VF		4 (33.3%)	4 (57.1%)	0 (0.0%)	
	Induced AT		1 (8.3%)	0 (0.0%)	1 (20.0%)	
	Induced AVRT		2 (16.7%)	1 (14.3%)	1 (20.0%)	
	Echocardiography∗		39 (100%)	25 (100%)	14 (100%)	1.000
	Mitral regurgitation		4 (10.3%)	3 (12.5%)	1 (7.1%)	1.000
	LV dysfunction		5 (12.8%)	4 (16.7%)	1 (7.1%)	.734
	Cardiac MRI∗		22 (56.4%)	12 (48.0%)	10 (71.4%)	.491
	LV dysfunction		2 (9.1%)	2 (16.7%)	0 (0%)	.542
	Gene test∗		20 (51.3%)	11 (44.0%)	9 (64.3%)	.378
	Benign		2 (10.0%)	1 (9.1%)	1 (11.1%)	.525
	Negative		7 (35.0%)	4 (36.4%)	3 (33.3%)	
	VUS		11 (55.0%)	6 (54.5%)	5 (55.6%)	

Comparisons were made between the IVF and ERS groups, and *P* values were calculated.

AF = atrial fibrillation; AT = atrial tachycardia; AVRT = atrioventricular reentrant tachycardia; ECG = electrocardiogram; ERS = early repolarization syndrome; IVF = idiopathic ventricular fibrillation; LV = left ventricular; MRI = magnetic resonance imaging; PVC = premature ventricular contraction; QTc = corrected QT interval; SVT = supraventricular tachycardia; VF = ventricular fibrillation; VT = ventricular tachycardia; VUS = variant of uncertain significance.

∗ Diagnostic tests performed in each group. The rows below each test indicate the corresponding results.

### Diagnostic assessment

ECG was performed in all patients (Table 1). No patient exhibited QT prolongation, T-wave abnormalities, pathologic Q waves, or ST-T changes suggestive of an acute coronary event. Holter (69.2%), treadmill test (56.4%), and drug testing (61.5%) (Supplemental Table 1) were performed in a limited number of patients, with no significant group differences. No patients showed a type 1 Brugada pattern during flecainide testing. An electrophysiological study was performed in 12 patients (30.8%); VF was inducible in 4 of 7 patients with IVF, but none of 5 patients with ERS. 1 patient with ERS had inducible ventricular tachycardia; overall ventricular arrhythmia inducibility showed no significant group difference (*P* = .488).

Echocardiography revealed no congenital coronary anomalies or structural heart diseases. Mild mitral regurgitation (4 [11.1%]) and left ventricular dysfunction (5 [13.9%]) resolved during follow-up, except in 2 patients lost to follow-up before repeat echocardiography. Cardiac magnetic resonance imaging (MRI) was performed in 22 patients (56.4%), with no late gadolinium enhancement. 2 patients with IVF showed mild left ventricular dysfunction on MRI despite normal echocardiography; follow-up MRI was not performed, but subsequent echocardiography confirmed normal function in both patients.

Genetic testing was performed in 20 patients (51.3%); 15 underwent next-generation sequencing panels, 4 underwent Sanger sequencing, and 1 underwent whole-exome sequencing. 7 patients (35.0%) had negative findings, 2 (10.0%) had benign variants, and 11 (55.0%) had variants of uncertain significance (Supplemental Table 2).

### ERP analysis in patients with ERS

Definitive ERP was not always evident at the time of the index VF event and was identified at a median of 8 days (0.5–63.5) after the index VF event (Supplemental Table 3). Among the 13 patients with ERS who experienced LAEs, ERP preceded recurrence in 11 patients and followed recurrence in 2 patients. During follow-up, the median proportion of ECGs demonstrating ERP was 0.74 (IQR 0.31–0.81) within the first year after the index event and 0.51 (IQR 0.30–0.82) across the entire follow-up period. Jp and D2 exhibited a weak positive correlation (*r* = 0.38; *P* = .17) (Supplemental Figure 2), which was accentuated when D2 was normalized to R-R interval (*r* = 0.56; *P* = .038). Scatter plots of Jp and D2 according to ERP characteristics, along with patients’ LAE status and time to LAE, are presented in Supplemental Figure 3. Patients with inferolateral ERP had significantly longer D2 than those with inferior or lateral ERP (36.2 ± 6.9 vs 56.9 ± 13.7; *P* = .03) (Supplemental Table 4), with a greater difference after normalization by QRS duration (*P* = .001). Similarly, D2 was longer in patients with pause-dependent dynamicity (36.6 ± 7.8 vs 56.9 ± 13.5; *P* = .022). D2 showed no association with ST-segment slope or fQRS, and no ERP feature correlated with Jp. However, the ST-segment slope varied by ERP location; lateral ERP mostly showed upward ST-segment slopes, whereas inferior and inferolateral ERP had horizontal or downward slopes (*P* = .014).

### Treatment and outcomes

Antiarrhythmic drugs were administered to 29 patients (74.4%). Class I agents (quinidine [n = 6], flecainide [n = 2], and mexiletine [n = 1]) were prescribed only in the ERS group (*P* = .001) (Table 2). Among patients with ERS treated with quinidine, initiation was prompted by recurrent LAEs in 4 patients, whereas 2 received quinidine based on the clinical diagnosis of ERS despite no documented recurrence at the time; no patient received isoproterenol for acute events. The ERS group received more medication classes than the IVF group (mean 1.57 ± 0.94 vs 0.76 ± 0.60; *P* = .005). ICDs were implanted in 33 patients (84.6%), at a mean age of 16.1 ± 2.3 years, with no group differences in implantation rate, age, type, or pacing mode. Appropriate shocks were more frequent in the ERS group (84.6% vs 50.0%; *P* = .099), although not statistically significant. 1 ICD-related complication (skin infection) occurred in a patient with IVF.

**Table 2:** Treatment and outcomes of the study population

Variables		Total	IVF	ERS	*P* value
		(N = 39)	(n = 25)	(n = 14)	
Antiarrhythmic drug	Class I	7 (17.9%)	0 (0.0%)	7 (50.0%)	.001∗
	Class II	27 (69.2%)	17 (68.0%)	10 (71.4%)	1.000
	Class III	4 (10.3%)	2 (8.0%)	2 (14.3%)	.944
	Class IV	2 (5.1%)	0 (0.0%)	2 (14.3%)	.237
	Number of classes	1.05 ± 0.83	0.76 ± 0.60	1.57 ± 0.94	.005∗
ICD	ICD insertion	33 (84.6%)	20 (80.0%)	13 (92.9%)	.545
	Age at insertion	16.1 ± 2.3	16.6 ± 2.3	15.4 ± 2.3	.149
	Appropriate shock	21 (63.6%)	10 (50.0%)	11 (84.6%)	.099
	Inappropriate shock	8 (24.2%)	3 (15.0%)	5 (38.5%)	.262
	Shock storm	3 (10.0%)	1 (5.6%)	2 (16.7%)	.709
	ICD complication	1 (3.0%)	1 (5.0%)	0 (0.0%)	.667
Outcome	Alive	34 (87.2%)	21 (84.0%)	13 (92.9%)	
	SCA	2 (5.1%)	2 (8.0%)	0 (0.0%)	.546
	Follow-up loss	3 (7.7%)	2 (8.0%)	1 (7.1%)	
Follow-up duration (y)		5.0 ± 4.1	5.2 ± 4.8	4.7 ± 2.7	.719
Neurologic sequalae		3 (7.7%)	3 (12.0%)	0 (0.0%)	1.000
LAE		26 (66.7%)	13 (52.0%)	13 (92.9%)	.025∗
LAE event	ACA	4 (15.4%)	2 (15.4%)	2 (15.4%)	.384
	VT/VF	1 (3.8%)	0 (0.0%)	1 (7.7%)	
	ICD Shock	19 (73.1%)	9 (69.2%)	10 (76.9%)	
	SCA	2 (7.7%)	2 (15.4%)	0 (0.0%)	
LAE duration (y)		2.2 ± 3.1	2.9 ± 3.5	1.1 ± 1.3	.024∗

ACA = aborted cardiac arrest; ERS = early repolarization syndrome; ICD = implantable cardioverter-defibrillator; IVF = idiopathic ventricular fibrillation; LAE = life-threatening arrhythmic event; SCA = sudden cardiac arrest; VT/VF = ventricular tachycardia/fibrillation.

∗ Significant associations.

**Figure 3:**
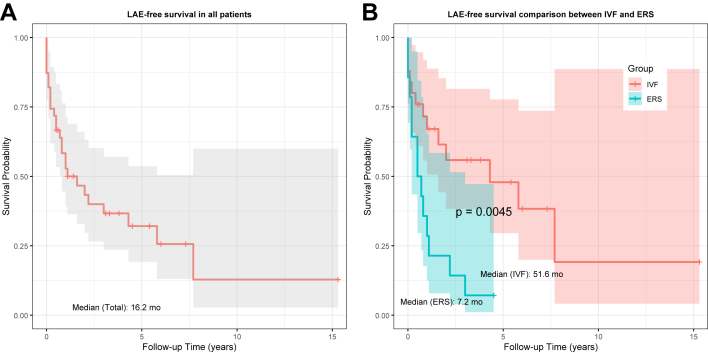
Kaplan–Meier survival curves of the study cohort. **A:** Overall survival of the total cohort including both ERS and patients with IVF. **B:** Comparison of survival between patients with ERS and patients with IVF. ERS = early repolarization syndrome; IVF = idiopathic ventricular fibrillation; LAE = life-threatening arrhythmic event.

During a mean follow-up of 5.0 ± 4.1 years, 2 patients (both IVF) died, and 3 were lost to follow-up. Neither deceased patient had an ICD implantation; 1 died of bleeding after ECMO for refractory VF, and the other, who had severe neurologic sequelae after the index VF, died of SCA 7.7 years later. Neurologic complications occurred in 3 patients—2 deceased and 1 lost to follow-up. LAEs occurred more frequently in the ERS group (92.9% vs 52.0%; *P* = .025). Median LAE-free survival was 16.2 months, significantly shorter in the ERS group (7.2 vs 51.6 months; *P* = .0045) (Figure 3).

### Prognostic factors

**Figure 4:**
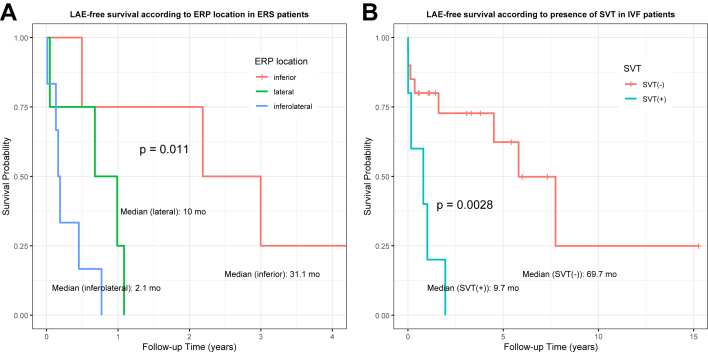
Kaplan–Meier survival curves of the study cohort. **A:** Survival in patients with ERS according to the location of early repolarization (inferior, inferolateral, or lateral leads). **B:** Survival in patients with IVF according to the presence of SVTs during follow-up. Median survival times are indicated with *dashed lines*. *P* values were calculated using the log-rank test. ERP = early repolarization pattern; ERS = early repolarization syndrome; IVF = idiopathic ventricular fibrillation; LAE = life-threatening arrhythmic event; SVT = supraventricular tachycardia.

In the ERS group, univariate analysis identified family history, Jp, and ERP location as risk factors for LAE (Table 3). Multivariable analysis showed only inferolateral ERP was significant (hazard ratio [HR] 9.29 [1.68–101.36], *P* = .009, vs inferior; HR 4.04 [0.80–26.89], *P* = .091, vs lateral). Kaplan–Meier curves confirmed the worst prognosis for inferolateral ERP, followed by lateral and inferior ERP (Figure 4A). Most known prognostic factors were not significant, except for a weak trend for a family history of SCA (*P* = .14) (Supplemental Figure 4).

**Table 3:** Univariable and multivariable regression for factors associated with life-threatening arrhythmic event-free survival in patients with early repolarization syndrome and idiopathic ventricular fibrillation

Variable	Early repolarization syndrome			
	Univariable hazard ratio (95% CI)	*P* value	Multivariable hazard ratio (95% CI)	*P* value
Age at presentation	0.96 (0.75–1.23)	.745		
Family history∗	2.40 (0.72–8.04)	.156†	1.46 (0.35–6.42)	.601
Jp	0.09 (0.003–2.75)	.168†	0.19 (0.00–47.26)	.542
Fragmented QRS	0.73 (0.24–2.19)	.572		
QRS duration	1.007 (0.97–1.04)	.681		
ERP location		.040†		.004†
Inferior	Ref.		Ref.	
Lateral	6.39 (0.70–58.67)	.101†	2.30 (0.31–30.90)	.432
Inferolateral	19.85 (1.89–208.44)	.013†	9.29 (1.68–101.36)	.009†
D2	1.03 (0.98–1.08)	.301		
ST-segment slope		.201		
Upward	Ref			
Horizontal/downward	0.46 (0.14–1.51)	.201		
Dynamicity	1.26 (0.40–4.03)	.695		
SVT	2.77 (0.32–24.17)	.358		
LV dysfunction	2.05 (0.24–17.64)	.512		
ERP (%) ≤1 y	0.84 (1.17–102.88)	.834		
ERP (%) total	0.62 (1.10–27.15)	.579		
Variable	Idiopathic ventricular fibrillation			
	Univariable hazard ratio (95% CI)	*P* value	Multivariable hazard ratio (95% CI)	*P* value
Age at presentation	0.92 (0.74–1.13)	.414		
Family history∗	0.82 (0.23–2.87)	.754		
Fragmented QRS	0.42 (0.13–1.32)	.138†	0.30 (0.08–1.17)	.081
LV dysfunction	1.65 (0.48–5.64)	.428		
SVT	5.54 (1.57–19.51)	.008†	7.24 (1.89–29.73)	.005†
Provocation by resting (vs others)	0.24 (0.03–1.81)	.176†	0.67 (0.07–3.42)	.661

NA indicates that the *P* value could not be calculated because of a lack of sufficient variability or data within the group. ERP (%): percentage of electrocardiograms demonstrating early repolarization pattern during the specified period (≤1 year after index VF or total follow-up).

CI = confidence interval; ERP = early repolarization pattern; LV = left ventricular; Ref. = reference; SVT = supraventricular tachycardia.

∗ Family history of sudden cardiac death or aborted cardiac arrest.

† Significant associations.

In the IVF group, SVT was associated with higher LAE risk in both univariable (HR 5.54 [1.57–19.51]; *P* = .008) and multivariable analyses (HR 7.24 [1.89–29.73]; *P* = .005). Kaplan–Meier curves confirmed poorer prognosis in patients with SVT(+) (Figure 4B). LAE-free survival by fQRS, age, and family history of SCA is presented in Supplemental Figure 5. Median LAE-free survival could not be determined in patients aged >16 years owing to low event rates, suggesting a possible trend toward better outcomes (*P* = .41).

## Discussion

### Distinguishing ERS from IVF: Diagnostic pitfalls

ERS is increasingly recognized as a distinct entity, not a subset of IVF.^7^^,^^8^ Our findings support this view; patients with ERS had a higher LAE incidence and poorer LAE-free survival, consistent with previous studies.^9^^,^^10^ This highlights the need for accurate differentiation to guide treatment decisions. Quinidine is recommended for patients with ERS and recurrent VF, and intravenous isoproterenol for acute management of electrical storms.^7^^,^^11^ Long-term antibradycardia pacing may be considered in bradycardia-triggered VF during sleep or rest.^12^

Despite its significance, distinguishing ERS from IVF remains challenging. In our study, 10 patients were initially misdiagnosed owing to unrecognized ERP. J-point elevations from hypothermia or myocardial injury after VF can mimic ERP. Limited clinician awareness also contributes to under-recognition. Conversely, ERP may be absent during the index event, even in patients later diagnosed as having ERS (Supplemental Table 3). These factors highlight the need for meticulous evaluation of serial ECGs.

### ERP features and their interrelationships

Various ERP features seemed to be interrelated. In particular, D2 was positively associated with Jp, ERP location, and dynamicity. Notably, a correlation between D2 and Jp was observed both across patients and within individuals over time. For example, serial ECGs from the same patient (Supplemental Figure 6) demonstrated that an ECG with longer D2 showed higher Jp amplitude. A similar within-individual relationship was observed for ERP location (Supplemental Figure 7), where the ECG with longer D2 demonstrated ERPs more clearly in multiple leads. These findings suggest that a more overt ERP (greater Jp) may coincide with broader spatial expression and greater temporal dispersion. Given that we selected the ECG with the most prominent ERP for each patient, these associations may reflect intraindividual variability and the dynamic nature, rather than fixed interindividual traits. Further studies in larger cohorts are needed to confirm these findings.

The relationship between ERP location and ST-segment slope has not been previously reported. Although both are known independent risk factors for VF^7^ in patients without prior VF, our cohort showed that inferior ERP consistently exhibited horizontal or downward slopes, whereas lateral ERP exhibited upward slopes. This suggests that these features may not be entirely independent in high-risk populations. Although limited by a small sample size, the co-occurrence of inferolateral J wave and a horizontal/downward ST-segment slope may represent a more specific VF risk factor.

### Age effect in the prognosis of IVF and ERS

Consistent with previous studies reporting poorer prognosis in younger patients than adults,^13^, ^14^, ^15^ our cohort showed a high incidence of LAE and short median LAE-free survival. Patients with IVF aged ≤16 years had a higher LAE rate (70% vs 40% in >16 years), consistent with Conte et al.^13^ Although not statistically significant in our cohort (Table 3, Supplemental Figure 5B), likely owing to the limited age range (all <19 years) and small sample size, this trend may be significant in a broader age range.

Age-related differences in ERS were less clear (Supplemental Figure 4F); LAE occurred in 2 of 5 patients aged >16 years and 8 of 9 patients aged ≤16 years, but interpretation is limited by the small sample size. In the ERS group, factors such as ERP location may outweigh the prognostic value of age. Nevertheless, combined with other studies showing a higher VF risk in younger patients with ERS^16^ and a lower LAE rate in adult ERS cohorts (36.4% VF recurrence rate),^17^ younger age may be associated with worse prognosis.

### Risk stratification in ERS

Most previous ERP studies assessed VF risk in individuals without prior VF,^16^^,^^18^^,^^19^ whereas we focused on patients with ERS with documented VF. Haruta et al^19^ reported that inferolateral ERP increased the risk of unexpected death, and expert consensus^7^ suggests that broader J-wave distribution implies worse prognosis. Similarly, our study found that inferolateral ERP was linked to poorer outcomes than localized ERP, suggesting that a wider distributed ERP reflects more extensive pathology and higher arrhythmic risk.

Other proposed markers of ERS such as fQRS, ST-segment slope, dynamicity, and family history were not prognostic in our cohort, possibly because previous VF overshadowed their effects. ST-segment slope showed a trend toward prognostic relevance in univariable analysis but did not reach statistical significance (*P* = .201), which may be explained by its close association with ERP location and the limited sample size. Therefore, the prognostic value of ST-segment slope cannot be excluded in larger cohorts.

We additionally assessed whether the temporal prevalence of ERP on serial ECGs—expressed as the proportion of ECGs demonstrating ERP during follow-up—was associated with recurrent arrhythmic events. Neither the 1-year nor the overall ERP proportion showed a significant association with outcomes. We also found no consistent perievent change (disappearance/re-emergence or accentuation) around LAE recurrence. However, interpretation is limited by heterogeneity in ECG acquisition frequency and clinical conditions at the time of recording (eg, SVTs and medications). Therefore, although our findings do not support ERP prevalence as a prognostic marker, they do not fully exclude a potential role of time-dependent ERP expression.

Morita et al^17^ studied patients with ERS (ERP[+] with previous VF), identifying fQRS, QRS widening, and longer D2 as predictors of future VF. In our cohort, fQRS, QRS interval, and D2 were not significantly associated with outcomes. Notably, D2 was correlated with ERP location, Jp, and dynamicity, suggesting its theoretical relevance to LAEs. Measurement challenges (ie, difficulty in defining the ERP end, particularly in slurred morphologies) may have limited its significance. However, given that D2 represents a very short interval, small measurement errors can lead to substantial differences. Therefore, assessing the QRS interval or, despite some variability, ERP location may provide more reliable prognostic information.

### Risk stratification in IVF

Given that IVF encompasses a heterogeneous group of IVF cases, the observed association with SVT may suggest a distinct IVF subgroup with broader electrical vulnerability extending beyond the ventricle. Other inherited arrhythmia syndromes, including catecholaminergic polymorphic ventricular tachycardia^20^^,^^21^ and congenital long QT syndrome,^22^^,^^23^ are recognized for having electrical instability that may involve both atrial and ventricular myocardium. In Brugada syndrome, atrial arrhythmias have been associated with an increased risk of ventricular arrhythmias or even SCA,^24^ supporting the concept that supraventricular arrhythmias may reflect a more extensive arrhythmogenic substrate. Taken together, although the prognostic significance of SVT in IVF remains speculative, our findings raise the possibility that SVT may serve as a marker of a higher-risk phenotype within IVF.

Although fQRS was proposed as a risk marker for IVF without ERP,^25^ it showed a paradoxical protective trend in our cohort. This may reflect follow-up limitations, given that 2 patients without fQRS died early or were lost to follow-up and may have later developed fQRS.

### Limitations

The small sample size and retrospective design restrict the generalizability of our findings. In addition, incomplete and nonuniform implementation of recommended diagnostic tests^4^ across centers may have limited the systematic exclusion of alternative causes of VF. For example, cardiac CT was not included in the original cohort protocol, limiting direct evaluation of ischemic heart disease. However, given its rarity in the pediatric population, the absence of ischemic ECG changes, lack of regional wall motion abnormalities, and recovery from transient ventricular dysfunction, ischemic heart disease is unlikely in this cohort. In this context, differentiation of distinct primary electrical disorders such as short-coupled VF was also limited, given that systematic analysis of ICD electrograms was not incorporated into the study protocol.

Furthermore, comprehensive risk modeling was constrained by the limited availability of uniformly collected candidate variables. In particular, detailed Holter-derived information—including morphologic characteristics and coupling intervals of premature ventricular contractions—was not systematically collected across centers. Electrophysiological studies were also performed using nonuniform protocols. As a result, multivariable models were limited to a small number of factors.

Finally, given that this study was conducted in a nationwide Korean pediatric cohort, population-specific genetic factors may have influenced ECG phenotypes and associated risk profiles. Accordingly, caution is warranted when extrapolating these findings to pediatric populations of other ethnic backgrounds.

## Conclusion

This study highlights distinct clinical features and prognostic factors of IVF and ERS, including outcome differences between pediatric and adult patients. Accurate differentiation between IVF and ERS may aid risk stratification and tailored management. Careful evaluation of ERP characteristics may be critical for risk stratification in patients with ERS. The poorer outcomes in pediatric patients underscore the need for increased clinical vigilance.

## Disclosures

The authors have no conflicts of interest to disclose.

## Footnotes

Appendix Supplementary data associated with this article can be found in the online version at https://doi.org/10.1016/j.hroo.2026.02.016.
