# Supplementary material for: Clinical manifestations and outcomes of idiopathic ventricular fibrillation and early repolarization syndrome in adolescents: A multicenter cohort study
Source: Heart Rhythm O2. 2026 Feb 24;7(5):959–67. doi: 10.1016/j.hroo.2026.02.016 (PMC13198355; doi:10.1016/j.hroo.2026.02.016)
Supplement: Supplementary Material [file mmc1.docx]

|  | **Total** | **IVF** | **ERS** | **p** |
| --- | --- | --- | --- | --- |
|  | (N=39) | (N=25) | (N=14) |  |
| **Drug test** | 24(61.5%) | 15 (60.0%) | 9 (64.3%) | 1 |
| **Flecainide** | 13(33.3%) | 9 (36.0%) | 4 (28.6%) | 0.906 |
| QT prolongation | 1(7.7%) | 0(0.0%) | 1(25.0%) | 0.070 |
| NSVT | 1(7.7%) | 0(0.0%) | 1(25.0%) |  |
| None | 11(84.6%) | 9(100.0%) | 2(50.0%) |  |
| **Epinephrine** | 12(30.8%) | 8 (32.0%) | 4 (28.6%) | 1.000 |
| QT prolongation | 6(50.0%) | 4(50.0%) | 2(50.0%) | 1 |
| None | 6(50.0%) | 4(50.0%) | 2(50.0%) |  |
| **Lidocaine** | 1(2.6%) | 0(0.0%) | 1 (7.1%) | 0.810 |
| PVC | 1(100.0%) | 0(0.0%) | 1(100.0%) |  |
| **isoproterenol** | 9(23.1%) | 6 (24.0%) | 3 (21.4%) | 1 |
| PVC | 1(11.1%) | 1(16.7%) | 0 (0%) | 1 |
| none | 8(88.9%) | 5(83.3%) | 3(100.0%) |  |
| **procainamide** | 1(2.6%) | 1 (4.0%) | 0 (0.0%) | 1 |
| none | 1(100.0%) | 1(100.0%) | 0 (0.0%) | 1 |

**Supplementary Table 1.** Drug Provocation Test Results in the Total Cohort and Subgroups (IVF vs. ERS)

This table summarizes the use and outcomes of drug provocation tests performed in the total study population (N = 39), and in subgroups of patients diagnosed with idiopathic ventricular fibrillation (IVF, N = 25) and early repolarization syndrome (ERS, N = 14).

| **Group** | **Method** | **Genes and Variants [Classification]** |
| --- | --- | --- |
| IVF | NGS Panel | HCN4 (c.2796C>T, p.Gly932) [VUS] TMEM43 (c.658C>T, p.Arg220Cys) [VUS] PRRT2 (c.236C>T, p.Ser79Leu) [VUS] |
|  | NGS Panel | KCNE3 (c.10A>G, p.Thr4Ala) [VUS] SLMAP (c.862C>T, p.His288Tyr) [VUS] |
|  | NGS Panel | RYR2 (c.11144A>G, p.Glu3715Gly) [VUS] |
|  | NGS Panel | PKP2 (c.2021T>C, p.Val674Ala) [VUS] RYR2 (c.538G>T, p.Asp180Tyr) [VUS] RYR2 (c.13175A>G, p.Lys4392Arg) [VUS] |
|  | NGS Panel | ROR2 (c.1885G>A, p.Val629Met) [VUS] DMD (c.2291A>G, p.Asn764Ser) [VUS] |
|  | NGS Panel | TRPM4 (c.571delT, p.Trp191fs) [VUS] |
|  | Sanger Sequencing | KCNH2 (c.3021C>T, p.Arg1007) [Benign] |
| ERS | NGS Panel | ACTN2 (c.784-452T>C) [VUS] |
|  | NGS Panel | FOXC1 (c.1063C>T, p.Pro3555Ser) [VUS] CACNA1C (c.5603_5605delCGAinsTGG, p.ProMet1868LeuVal) [VUS] TTN (c.39230T>C, p.Val13077Ala) [VUS] TTN (c.19995A>T, p.Glu6665Asp) [VUS] |
|  | NGS Panel | SCN5A (c.5851G>T, p.Val195Leu) [VUS] SNTA1 (c.497-7C>T, splicing) [VUS] AKAP9 (c.5495G>A, p.Gly1832Glu) [VUS] |
|  | NGS Panel | AGRN (c.5501C>T, p.Pro1834Leu) [VUS] NEB (c.2431G>A, p.Asp811Asn) [VUS] CHRNA1 (c.1244C>T, p.Ala415Val) [VUS] TTN (c.17545A>G, p.Thr5849Ala) [VUS] DAG1 (c.220G>A, p.Val74Ile) [VUS] |
|  | WES | ANK2 (c.6725C>T, p.Thr2242Met) [Benign] |

**Supplementary Table 2.** Summary of Genetic Variants Identified in IVF and ERS Patients. Genetic variants identified in patients with idiopathic ventricular fibrillation (IVF) and early repolarization syndrome (ERS). Each row represents a single patient. Variant classification is based on ACMG guidelines.

NGS = next-generation sequencing; WES = whole-exome sequencing.

| ID | Jp (mV) | | | Jt (mV) | | | D1 (msec) | | | D2 (msec) | | | 1^st^ ERP day | ERP (%),  ≤1 yr | ERP (%), total | Location | ST slope | Dyn | fQRS |
| --- | --- | --- | --- | --- | --- | --- | --- | --- | --- | --- | --- | --- | --- | --- | --- | --- | --- | --- | --- |
|  | R1 | R2 | Mean | R1 | R2 | Mean | R1 | R2 | Mean | R1 | R2 | Mean |  |  |  |  |  |  |  |
| 1 | 0.29 | 0.30 | 0.30 | 0.043 | 0.043 | 0.043 | na | na | na | 33.4 | 34.0 | 33.7 | na | na | 100% | Inf | Hor | na | + |
| 2 | 0.23 | 0.23 | 0.23 | 0.061 | 0.066 | 0.063 | 11.0 | 16.1 | 13.5 | 34.6 | 39.8 | 37.2 | 179 | 7% | 17% | Lat | up | - | + |
| 3 | 0.11 | 0.11 | 0.11 | 0.023 | 0.035 | 0.029 | 9.2 | 6.3 | 7.8 | 42.1 | 40.3 | 41.2 | 767 | 0% | 11% | Lat | up | - | - |
| 4 | 0.73 | 0.68 | 0.70 | 0.062 | 0.006 | 0.034 | 11.0 | 11.5 | 11.2 | 47.3 | 51.9 | 49.6 | 3 | 100% | 100% | Inf | down | + | + |
| 5 | 0.40 | 0.41 | 0.40 | 0.213 | 0.231 | 0.222 | 11.0 | 12.1 | 11.5 | 32.9 | 34.6 | 33.7 | 7 | 74% | 61% | Lat | up | - | + |
| 6 | 0.35 | 0.35 | 0.35 | 0.078 | 0.086 | 0.082 | na | na | na | 32.9 | 33.4 | 33.1 | 0 | 80% | 76% | Inf & Lat | up | - | - |
| 7 | 0.28 | 0.27 | 0.27 | 0.078 | 0.086 | 0.082 | na | na | na | 52.4 | 49.0 | 50.7 | 0 | 100% | 100% | Inf & Lat | down | - | - |
| 8 | 0.51 | 0.48 | 0.49 | 0.033 | 0.04 | 0.037 | 19.0 | 17.3 | 18.2 | 74.9 | 69.2 | 72.0 | 9 | na | na | Inf & Lat | up | + | - |
| 9 | 0.21 | 0.18 | 0.20 | 0.108 | 0.101 | 0.104 | 14.4 | 17.3 | 15.9 | 33.4 | 34.6 | 34.0 | 15 | na | na | Lat | up | + | + |
| 10 | 0.43 | 0.42 | 0.42 | 0.065 | 0.065 | 0.065 | 15.6 | 20.2 | 17.9 | 78.4 | 46.1 | 62.2 | 0 | 47% | 34% | Inf & Lat | down | + | - |
| 11 | 0.53 | 0.53 | 0.53 | 0.048 | 0.127 | 0.087 | 15.0 | 16.1 | 15.6 | 57.6 | 73.2 | 65.4 | 0 | 82% | 52% | Inf & Lat | down | + | + |
| 12 | 0.18 | 0.16 | 0.17 | 0.022 | 0.014 | 0.018 | 8.1 | 8.6 | 8.4 | 25.9 | 25.9 | 25.9 | 12 | 40% | 50% | Inf | Hor | - | + |
| 13 | 0.58 | 0.58 | 0.58 | 0.069 | 0.063 | 0.066 | 7.5 | 6.9 | 7.2 | 31.7 | 36.9 | 34.3 | 112 | 78% | 36% | Inf | down | - | - |
| 14 | 0.25 | 0.27 | 0.26 | 0.069 | 0.072 | 0.071 | 17.9 | 17.3 | 17.6 | 54.8 | 61.7 | 58.2 | 132 | 21% | 17% | Inf & Lat | down | + | - |
| ICC | 0.993 | | | 0.934 | | | 0.832 | | | 0.778 | | |  |  |  |  |  |  |  |

**Supplementary table 3**. Interobserver Measurements and Interclass Correlation Coefficient for ECG Parameters. Early repolarization amplitudes (Jp, Jt) and durations (D1, D2) measured by two independent raters in 14 patients, along with their mean values. Interobserver reliability was assessed using intraclass correlation coefficients (ICC). Additional clinical features including ERP day, ERP location, ST slope, dynamicity, and fragmented QRS (fQRS) presence are also shown.

R1 = S.M.B (Rater 1), R2 = M.K.S (Rater 2)

Hor = horizontal, Lat = lateral, Inf = inferior, Inf&Lat = inferolateral, up = upward, down = downward

1^st^ ERP day = Days from initial VF event to first observation of early repolarization pattern on ECG.

ERP (%): percentage of ECGs demonstrating early repolarization pattern during the specified period (≤1 year after index VF or total follow-up).

Dyn (dynamicity): presence of pause-dependent augmentation of ERP.

fQRS: fragmented QRS complex.

**Supplementary table 4** Comparison of ECG parameter and characteristics according to early repolarization pattern location and dynamicity in patients with early repolarization syndrome

|  | **Inferior** | **Lateral** | **Inferolateral** | P |
| --- | --- | --- | --- | --- |
|  | (N=4) | (N=4) | (N=6) |  |
| **D2 (ms)** | 35.9 ± 9.9 | 36.5 ± 3.5 | 56.9 ± 13.7 | 0.084 |
|  | 36.2 ± 6.9 | |  | **0.033** |
| **D2/QRSd** | 0.4 ± 0.1 | 0.4 ± 0.0 | 0.5 ± 0.1 | **0.012** |
|  | 0.4 ± 0.0 | |  | **0.001** |
| **Jp (mV)** | 0.4 ± 0.2 | 0.2 ± 0.1 | 0.4 ± 0.1 | 0.183 |
| **Dynamicity** | 1 (33.3%) | 1 (25.0%) | 4 (66.7%) | 0.380 |
|  | 2 (28.6%) | |  | 0.415 |
| **ST segment slope** |  | | | **0.014** |
| -down/horizontal | 4 (100.0%) | 0 (0.0%) | 4 (66.7%) |  |
| -upward | 0 (0.0%) | 4 (100.0%) | 2 (33.3%) |  |
|  | **Dynamicity (-)** | | **Dynamicity (+)** | P |
|  | (N=7) | | (N=6) |  |
| **D2 (ms)** | 36.6 ± 7.8 | | 56.9 ± 13.5 | **0.022** |
| **D2/QRSd** | 0.4 ± 0.0 | | 0.5 ± 0.1 | **0.022** |
| **Jp (mV)** | 0.3 ± 0.2 | | 0.4 ± 0.2 | 0.234 |
| **ST segment slope** |  | | | 0.321 |
| - down/horizontal | 3 (42.9%) | | 4 (66.7%) |  |
| - upward | 4 (57.1%) | | 2 (33.3%) |  |

D2, D2/QRSd, Jp, pause/bradycardia-dependent dynamicity, and ST-segment slope were compared among patients with inferior, lateral, and inferolateral J-wave (top section) and between patients with and without J-wave dynamicity (bottom section). Significant differences were observed in the D2, D2/QRSd, and ST-segment slopes according to the J-point location and dynamicity.

**
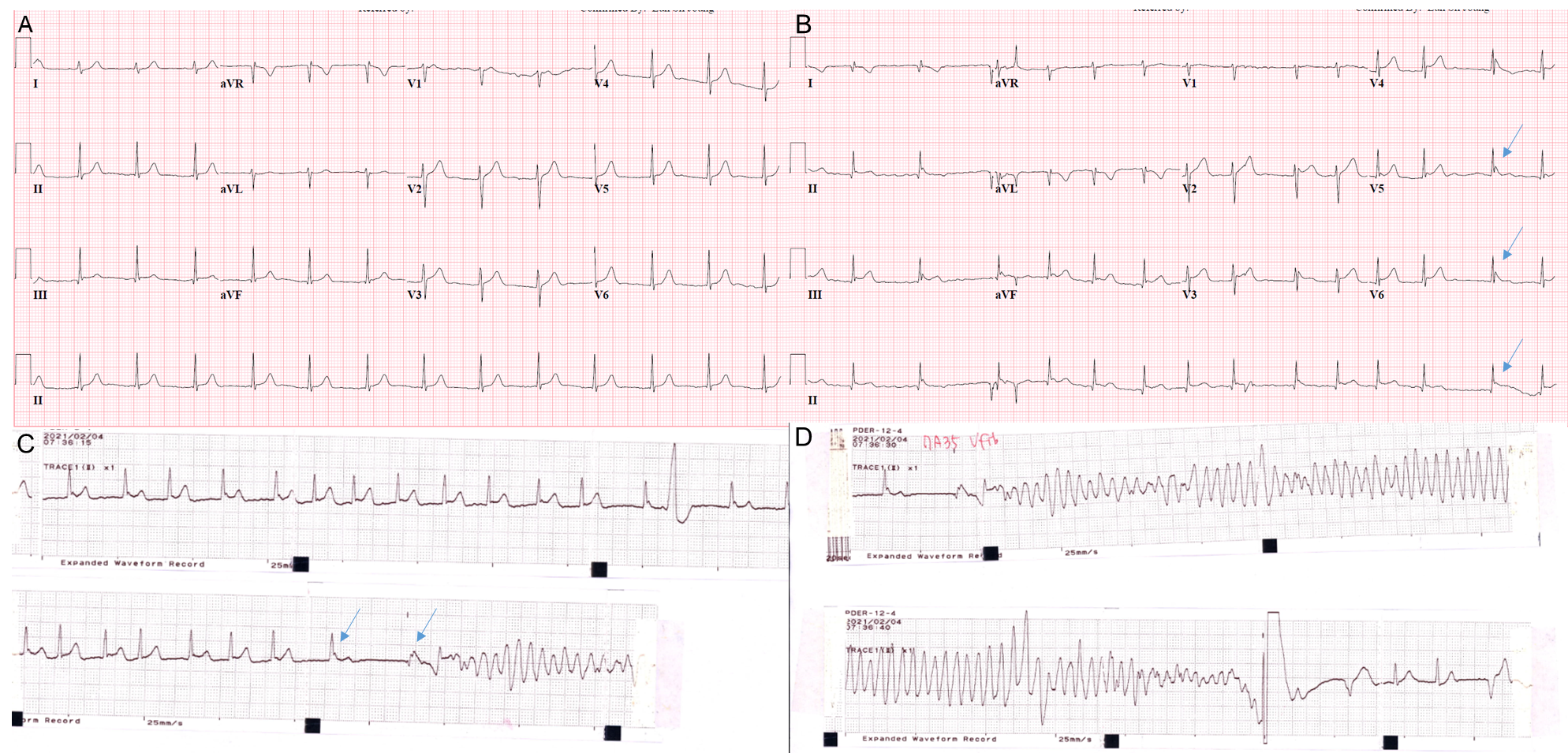
**

**Supplementary Figure 1**. A 14-years-old girl (patient ID 14) diagnosed with early repolarization syndrome and pause-dependent dynamicity, followed by VF

(A) Initial ECG at diagnosis showed normal sinus rhythm without early repolarization features. She was diagnosed with idiopathic VF and received an ICD implantation. (B) Eight months later, she developed paroxysmal atrial fibrillation with pause-dependent augmentation of the J wave (arrows). (C) Telemetry monitoring revealed progressive J wave augmentation following sinus pauses (blue arrows). (D) A prolonged pause led to marked J wave elevation followed by VF, which was successfully terminated by an ICD shock. Anti-bradycardia ventricular pacing effectively suppressed VF recurrence, and quinidine successfully prevented further atrial fibrillation episodes.


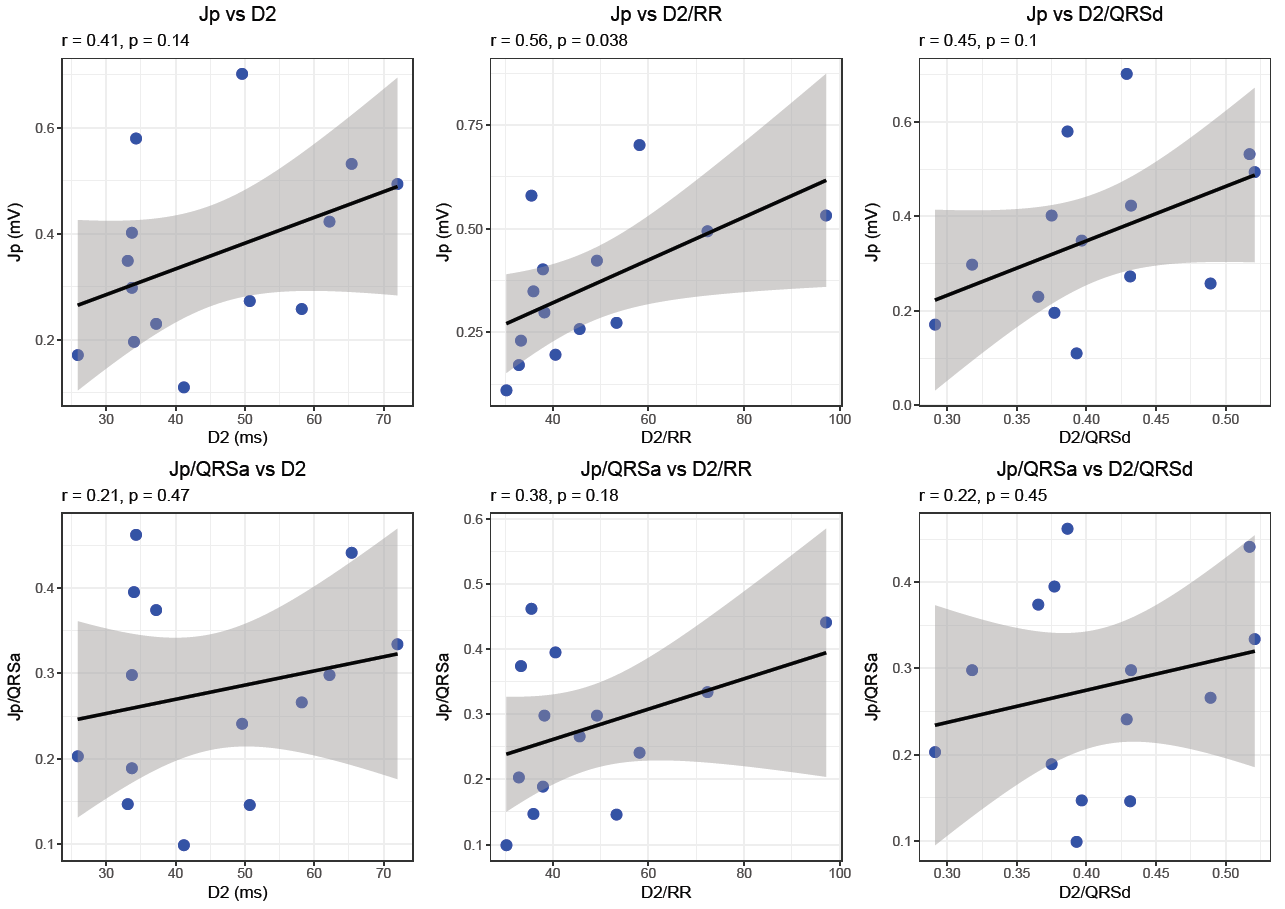


**Supplementary Figure 2**. Correlation between J-wave parameters and repolarization markers.

Scatter plots showing the relationship between J-point amplitude (Jp, top row) or Jp normalized by QRS amplitude (Jp/QRSa, bottom row) and duration factors: absolute D2 interval (left), D2 corrected by RR interval (middle), and D2 normalized by QRS duration (right). A significant positive correlation was observed between Jp and D2/RR (r = 0.56, p = 0.038)


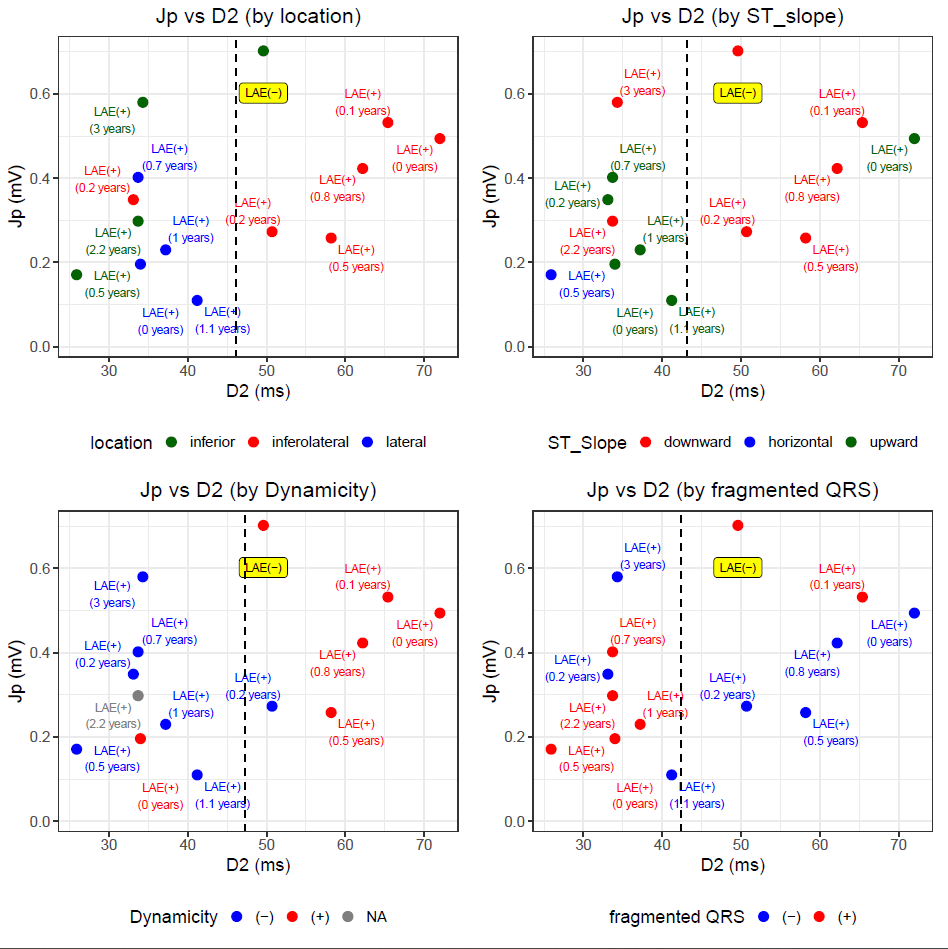


Supplementary **Figure 3**

Comparison of Jp and D2 interval according to ECG characteristics. Each scatter plot shows the relationship between Jp and D2 stratified by ECG features: (**A**) J-wave location (inferior, inferolateral, lateral), (**B**) ST-segment slope (upward, horizontal, downward), (**C**) pause/bradycardia-dependent dynamicity, and (**D**) presence of fragmented QRS. The dot color indicates the corresponding group, whereas the labels denote whether a life-threatening arrhythmic event (LAE) occurred and the follow-up time to LAE. The dashed line indicates the average of the median D2 values from the two groups (or the two most distinct groups in the three-group comparison), which served as a visual reference point for group differences.


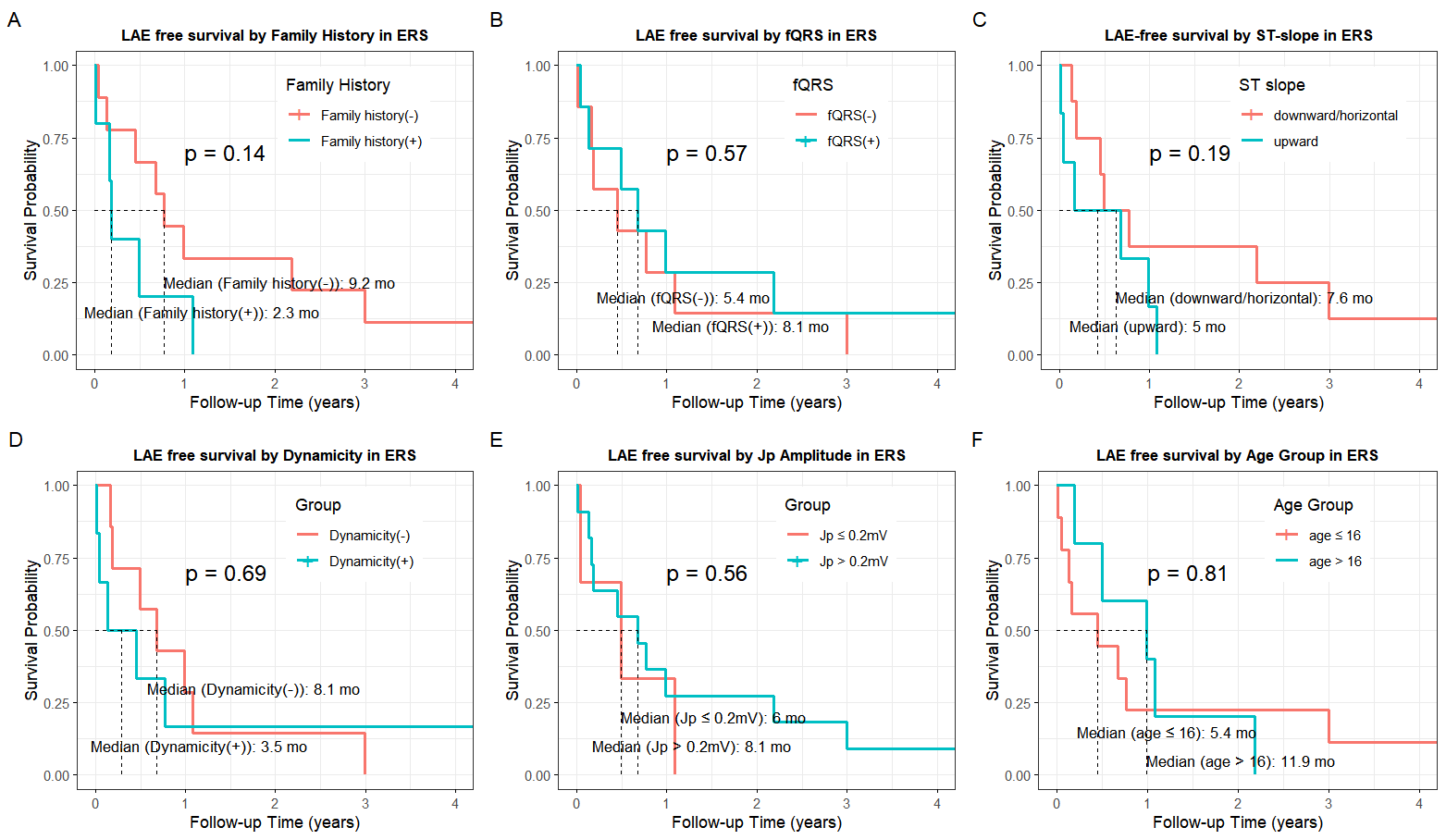


**Supplementary Figure 4**. Kaplan–Meier curves for LAE-free survival stratified by ECG features and demographic parameters in patients with early repolarization syndrome (ERS). (A) Family history of sudden cardiac arrest, (B) presence of fragmented QRS (fQRS), (C) ST-segment slope direction (downward/horizontal vs. upward), (D) pause dependent dynamicity of J-waves, (E) J-point amplitude (Jp ≤ 0.2 mV vs. > 0.2 mV), and (F) age group (≤ 16 years vs. > 16 years). Median LAE-free survival time is annotated for each group when estimable. No statistically significant differences were observed across groups (all p > 0.05).


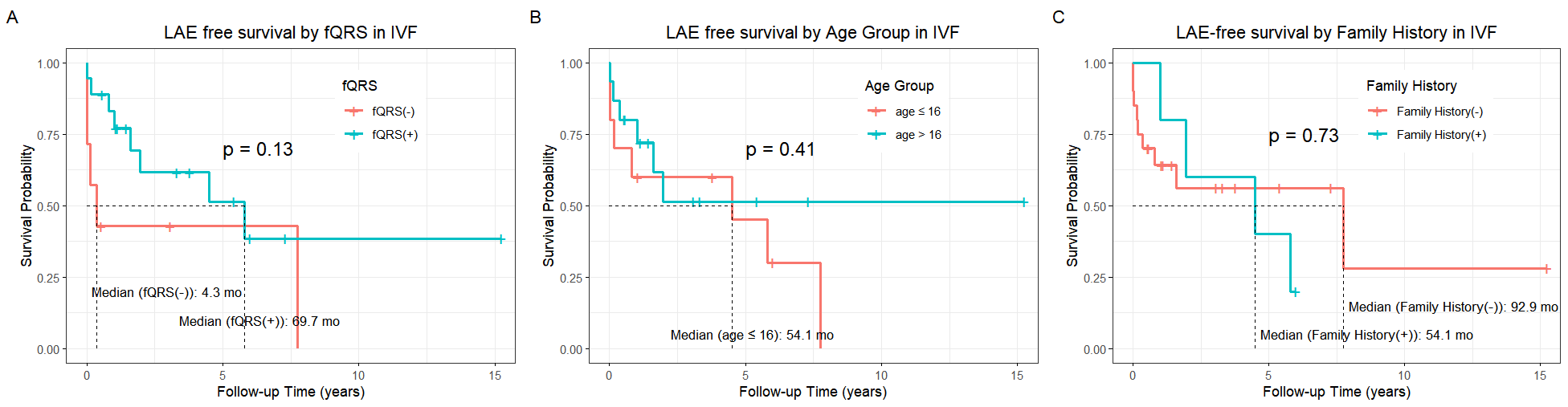


**Supplementary Figure 5**. Kaplan–Meier Survival Curves for LAE free survival stratified by ECG features and demographic parameters in patients with idiopathic ventricular fibrillation (IVF). (A) Presence of fragmented QRS (fQRS), (B) age at diagnosis (≤ 16 years vs. > 16 years), and (C) presence of family history of sudden cardiac arrest. Median LAE-free survival time is annotated for each group when estimable. No statistically significant differences were observed between the groups (p = 0.13 and p = 0.41, and p = 0.73, respectively)


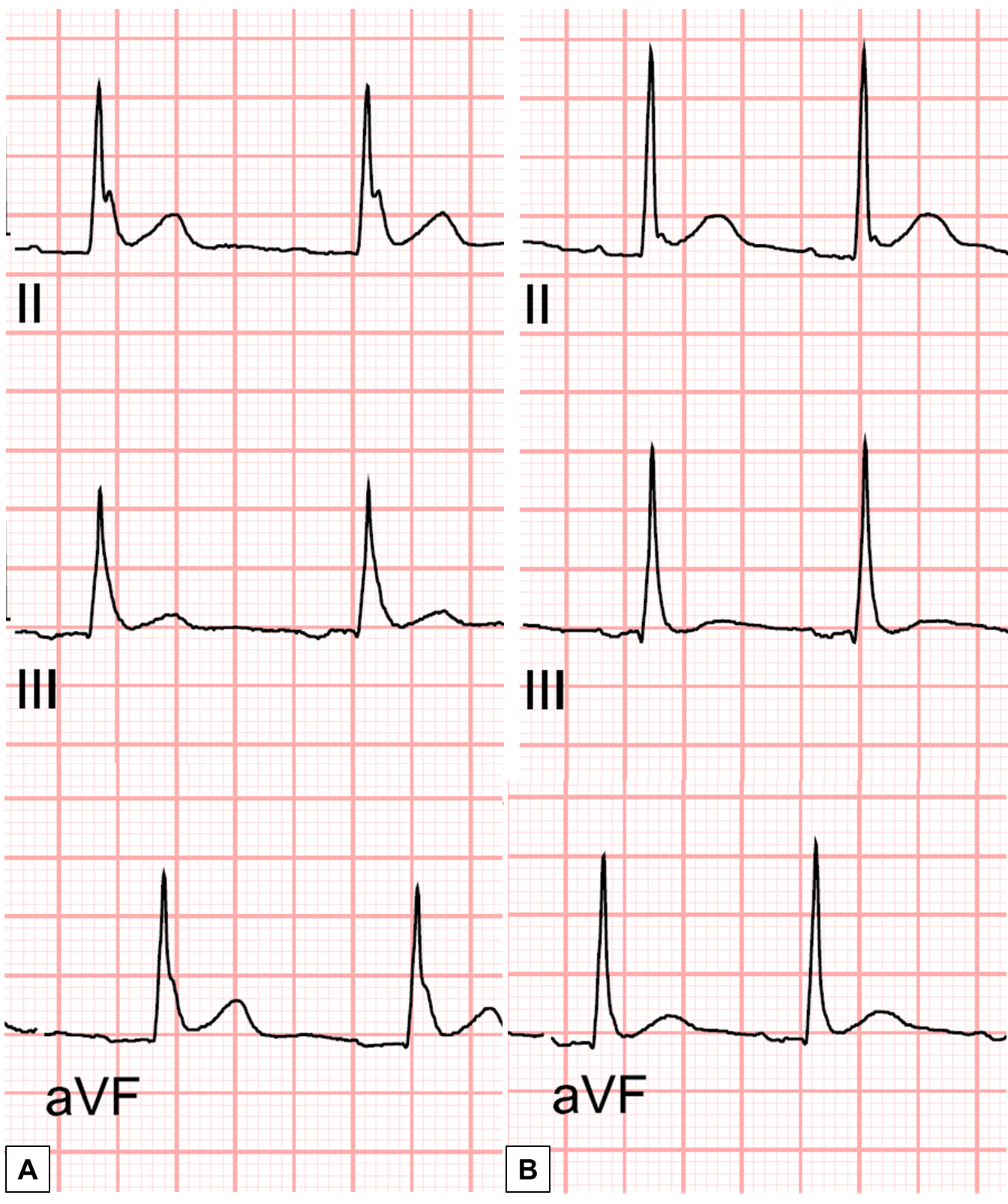


**Supplementary Figure 6.** Two ECGs obtained on different days from the same patient (Patient ID 8). (A) On the day with a longer D2 (57ms), the Jp in lead II is larger, and a more prominent slur is observed in lead III, aVF. (B) With shorter D2 (41ms) Jp is smaller in lead II, and the slurs in leads III, aVF appear more obscure.


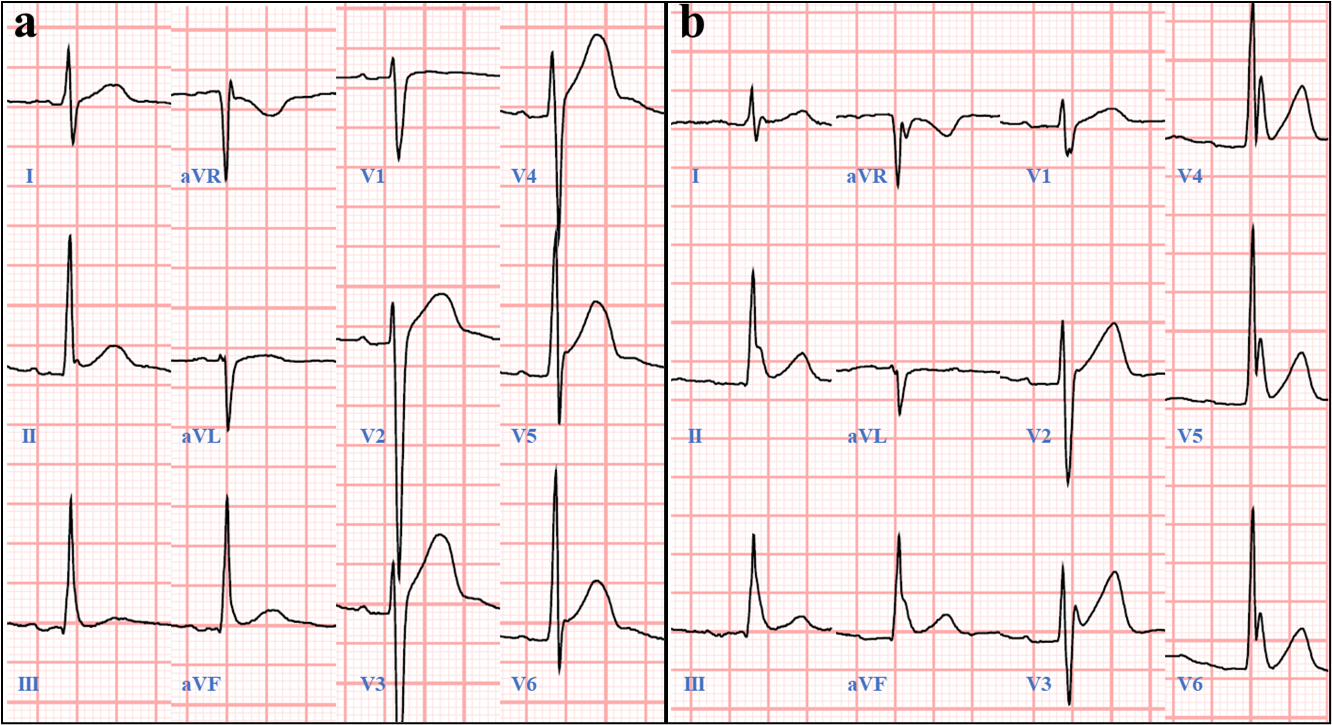


**Supplementary Figure 7.** Two electrocardiograms obtained on different days from the same patient (Patient ID 8). (A) ERP is visible only in the inferior leads, with only Jt elevation without notch or slur in lead V4-6. (B) ERP is observed not only in the inferior leads but also in leads V4-6, with longer D2 compared to panel A (45ms vs. 58ms, measured at aVF).
